# Pulmonary Artery Intimal Sarcoma: Diagnostic and Surgical Strategy from a Rare Case

**DOI:** 10.70352/scrj.cr.25-0098

**Published:** 2025-09-26

**Authors:** Takuro Hieda, Eijiro Nogami, Yasuhito Hosoda, Takahiro Miho, Jun Yanagisawa, Yuji Katayama

**Affiliations:** 1 Department of Cardiovascular Surgery, Fukuoka Tokushukai Hospital, Kasuga, Fukuoka, Japan; 2 Department of Surgery, Fukuoka Tokushukai Hospital, Kasuga, Fukuoka, Japan

**Keywords:** endovascular biopsy, radical surgery, pulmonary artery intimal sarcoma, cardiopulmonary bypass, median sternotomy

## Abstract

**INTRODUCTION:**

Pulmonary artery intimal sarcoma (PAIS) is a rare tumor, and the prognosis is extremely poor. Radical surgery for PAIS is the best option for prolonging survival. Thus, PAIS should be diagnosed as soon as possible, and radical surgery should be performed rapidly. However, it is difficult to differentiate PAIS from pulmonary embolism in the early stage. We report the case of a patient who was diagnosed with PAIS by endovascular catheter biopsy in the early period and who underwent left pneumonectomy, accompanied by resection of the main and right pulmonary arteries and right pulmonary artery reconstruction via median sternotomy for radical surgery.

**CASE PRESENTATION:**

A 53-year-old man presented to cardiologists for dyspnea. Contrast-enhanced CT revealed an occlusive mass in the left and main pulmonary arteries. The cardiologists diagnosed the patient with pulmonary embolism and started administering an anticoagulant. However, the mass did not shrink, and the cardiologist consulted the cardiovascular surgeons. A cardiologist performed an endovascular catheter biopsy to differentiate between the thrombus and the tumor. The biopsy specimen tissue contained atypical cells and was negative for Mouse Double Minute protein 2. The surgery was performed via median sternotomy. Cardiopulmonary bypass was established. The tumor filled the left pulmonary artery and extended to the main and right pulmonary arteries. With sufficient margins from the tumor, the main and right pulmonary arteries were resected and subjected to rapid intraoperative pathological diagnosis, with positive margins of the main pulmonary artery. Additional resection just above the pulmonary artery valve and reconstruction of the right pulmonary artery were performed, followed by left pneumonectomy. The postoperative histological diagnosis of the tumor was PAIS. The surgical margin of the main pulmonary artery was microscopically positive for tumor cells. Chemoradiotherapy was started postoperatively. There was no recurrence in the pulmonary artery, but a head MRI revealed a metastatic brain tumor, and the patient died approximately 1.5 years after surgery.

**CONCLUSIONS:**

Endovascular catheter biopsies are useful for differentiating thrombi from tumors. Furthermore, an early diagnosis of PAIS is important to ensure adequate time for discussing the surgical strategy with thoracic surgeons.

## Abbreviations

α-SMA
: alpha-smooth muscle actin
CPB
: cardiopulmonary bypass
FEV1.0
: forced expiratory volume in 1 second
MDM2
: Mouse Double Minute protein 2
MIB-1
: molecular immunology borstel antibody 1
PAIS
: pulmonary artery intimal sarcoma
PE
: pulmonary embolism
MSI
: microsatellite instability
TMB
: tumor mutation burden
VC
: vital capacity

## INTRODUCTION

PAIS is a rare tumor with an incidence of 0.01–0.03%.^1)^ The prognosis of PAIS is poor, and survival is usually 12–18 months from the time of diagnosis.^2)^ Mean survival of PAIS is <2 months in patients who do not undergo surgery.^2)^ The symptoms and imaging findings of PAIS are similar to those of PE, making differentiation difficult. The treatment for PAIS includes surgery, chemotherapy, radiotherapy, and heavy-ion radiotherapy; however, radical surgical resection is the best option for prolonged survival.^3)^ Herein, we report the case of a patient in whom PAIS and PE were differentiated via an endovascular catheter biopsy and who underwent left pneumonectomy accompanied by the resection of the main and right pulmonary arteries and reconstruction of the right pulmonary artery via median sternotomy.

## CASE PRESENTATION

**Fig. 1:**
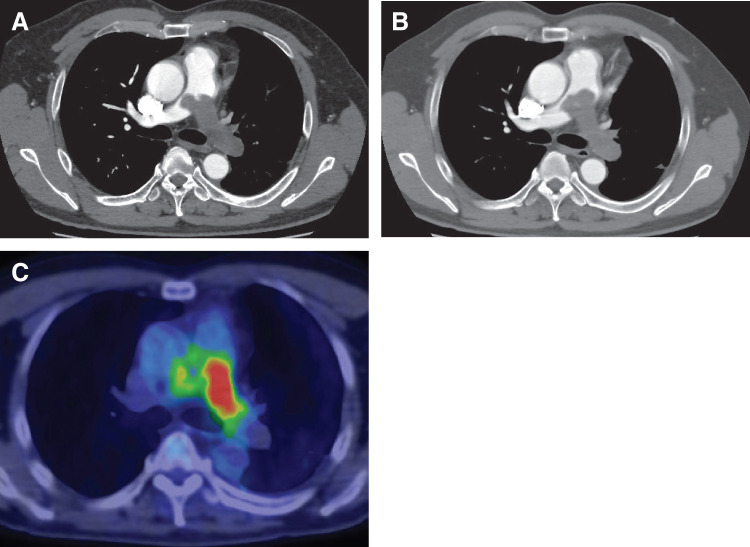
(**A**) Contrast-enhanced CT during the 1st examination shows that the mass filled the left pulmonary artery and extended to the main and right pulmonary arteries. (**B**) Contrast-enhanced CT after 10 days of anticoagulant therapy shows that the mass did not change in size. (**C**) PET-CT reveals high fluorodeoxyglucose accumulation with a maximum standardized uptake value of 13.38 in the left pulmonary artery. PET-CT, positron emission tomography-CT

A 53-year-old man presented to the Department of Cardiology with dyspnea. Contrast-enhanced CT revealed an occlusive mass in the left and main pulmonary arteries (**Fig. 1**). The cardiologists and radiologists diagnosed the patient with PE and prescribed anticoagulant therapy. However, the mass did not shrink after 10 days of treatment.

**Fig. 2:**
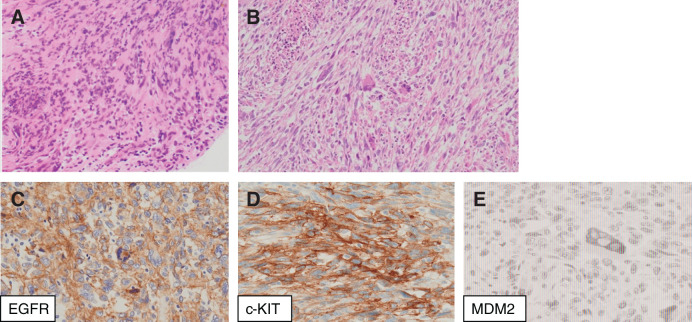
(**A**) Endovascular catheter biopsy showing atypical spindle-shaped cells. (**B**) Postoperative specimen showing the proliferation of atypical spindle and pleomorphic cells with necrosis. (**C**, **D**, and **E**) Immunological examination reveals positivity for EGFR and c-kit but negativity for MDM2. EGFR, epidermal growth factor receptor; MDM2, Mouse Double Minute protein 2

A physical examination revealed no abnormalities. Blood examination revealed an elevated D-dimer level of 1.0 μg, normal renal function, and no anemia or thrombocytopenia. Preoperative spirometry revealed a VC of 3320 mL (%VC: 80.2%) and a forced expiratory volume in 1 second (FEV1.0) of 2510 mL (FEV1.0%: 79.4%). Pulmonary artery catheterization revealed pulmonary hypertension (mean pulmonary artery pressure: 27 mmHg). PET-CT revealed high fluorodeoxyglucose accumulation with a maximum standardized uptake value of 13.38 in the left pulmonary artery (**Fig. 1**). Moreover, PET-CT revealed no metastasis. A cardiologist performed an endovascular catheter biopsy to differentiate between the thrombus and the tumor. Histological examination of the biopsy specimen revealed atypical spindle-shaped cells, and immunological examination revealed positivity for AE1/AE3, α-SMA, and c-kit, but negativity for CD68, CD3, CD20, desmin, CD34, and MDM2 (**Fig. 2**). The pathologists diagnosed the patient with PAIS, and we decided to perform surgery.

There was a high risk of sudden death because of the obstruction of the bilateral pulmonary artery, as the tumor rapidly increased in size. Therefore, we needed to perform the surgery as soon as possible to avoid sudden death. Additionally, we planned to perform a radical surgery because there was a sufficient surgical margin and PET-CT revealed no metastasis. The strategy for performing radical surgery was left pneumonectomy, accompanied by resection of the main and right pulmonary arteries and reconstruction of the right pulmonary artery.

**Fig. 3:**
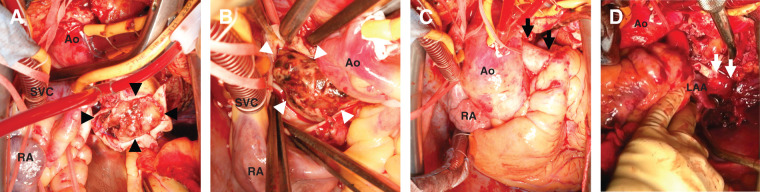
(**A**) Resection of the main pulmonary artery and visualization of the tumor (black arrowheads). The tumor appears to fill the left pulmonary artery. (**B**) The tumor and thrombus (white arrowheads) extend into the right pulmonary artery. (**C**) Reconstruction of the right pulmonary artery with an artificial blood vessel (black arrows). (**D**) Left pneumonectomy is performed via median sternotomy. The left inferior pulmonary vein (white arrows) was safely resected because the heart was collapsed under CPB with a left ventricular vent. Ao, aorta; CPB, Cardiopulmonary bypass; LAA, left atrial appendage; RA, right atrium; SVC, superior vena cava

The surgery was performed via median sternotomy. CPB was performed after inserting an arterial cannula into the ascending aorta and inserting venous cannulas into the superior and inferior vena cavae. The ascending aorta was clamped, and the main pulmonary artery was incised under cardiac arrest. The tumor filled the left pulmonary artery and extended into the main and right pulmonary arteries (**Fig. 3**). With a sufficient margin surrounding the tumor, the main and right pulmonary arteries were resected and subjected to a rapid intraoperative pathological diagnosis, with positive margins of the main pulmonary artery. Additional resection was performed immediately above the pulmonary artery valve, and the right pulmonary artery was reconstructed using artificial blood vessels. After the aorta was declamped, the Marshall ligament, Botallo’s ligament, and pericardium were incised, and the left pulmonary artery was dropped into the intrapleural space. Because the heart collapsed on-pump with the left ventricular vent, the left superior and inferior pulmonary veins and the left main bronchus were resected in the intrapleural space via median sternotomy, followed by left pneumonectomy. The operation, CPB, and aortic clamp times were 442, 292, and 149 min, respectively, and the blood loss volume was 1400 mL.

Macroscopically, the tumor filled the left pulmonary artery. Histopathological examination revealed the proliferation of atypical spindle and pleomorphic cells with necrosis. Immunological examination revealed positivity for the epidermal growth factor receptor, c-kit, α-SMA, and MIB-1, but negativity for the MDM2 (**Fig. 2**). A pathological diagnosis of PAIS was established. Finally, the surgical margin of the main pulmonary artery was found to be microscopically positive for tumor cells.

The patient’s postoperative course was uneventful. The patient was discharged from the hospital with home oxygen therapy on POD 12 because of shortness of breath without decreased arterial oxygen saturation during exercise. Radiotherapy (60 Gy in 30 fractions) at the surgical margin was initiated on POD 19, and three cycles of chemotherapy (ifosfamide, 2 mg/m^2^; doxorubicin, 30 mg/m^2^) were initiated on POD 117. Although there was no recurrence of PAIS in the pulmonary artery, the patient experienced diplopia and headaches. MRI revealed a metastatic brain tumor in the left midbrain and left parietal lobe on POD 317. Hydrocephalus secondary to a midbrain tumor was identified, and a ventriculoperitoneal shunt was placed on POD 325. In addition, radiotherapy (32 Gy in four fractions) was administered to the brain lesion. Follow-up MRI on POD 428 demonstrated a reduction in the size of the left midbrain tumor; however, the left parietal lobe lesion remained unchanged, and a new metastatic lesion had developed in the frontal lobe. Genetic panel testing revealed high MSI and TMB. However, no systemic chemotherapy was administered for the brain metastasis because of the deterioration in the performance status of the patient. Following a multidisciplinary discussion, the patient elected to pursue best supportive care, and the patient died on POD 508.

## DISCUSSION

PAIS is frequently misdiagnosed as PE owing to similar symptoms and imaging findings. The symptoms of patients with PAIS typically involve the cardiovascular or pulmonary systems and include dyspnea, syncope, and hemoptysis.^4)^ No specific symptoms are associated with PAIS. Moreover, contrast-enhanced CT findings of PAIS include “wall-eclipsing signs,” high-density lesions, uneven enhancement caused by hemorrhage, and filling defects with soft tissue features in the pulmonary artery.^4,5)^ However, differentiating PAIS from PE is difficult because these findings do not appear in the early stages. In the present case, the cardiologists and radiologists diagnosed the patient with PE and administered anticoagulants following the initial examination. However, the cardiologists did not prescribe anticoagulants for an extended period and performed an endovascular catheter biopsy to differentiate between thrombi and tumors. Early consideration of a pulmonary tumor likely contributed to the early diagnosis of PAIS.

A preoperative histological diagnosis of PAIS can be established using several methods, such as percutaneous biopsy under CT guidance, transbronchial biopsy, and endovascular catheter biopsy.^6)^ Among these methods, an endovascular catheter biopsy has been reported to be useful for confirming the diagnosis in a few case reports.^6,7)^ In a previous study, it was used to successfully detect PAIS in more than 75% of the patients.^8)^ However, the complications of this procedure include perforation, bleeding, pseudoaneurysm, and dissemination.^7)^ We recognized the risks of endovascular catheter biopsy but believed that a confirmed diagnosis had more benefits than these risks. These advantages include the consideration of a radical surgical strategy instead of thrombectomy to differentiate between thrombi and tumors. In this case, we needed to cooperate with thoracic surgeons to perform the radical surgery and could hold sufficient conferences on the surgical strategy. Lu et al. preoperatively misdiagnosed PAIS as a chronic PE and performed pulmonary artery endarterectomy.^9)^ However, the postoperative diagnosis was PAIS, and the patient died caused by tumor progression and recurrence.^9)^ Therefore, we suggest performing an endovascular catheter biopsy to detect tumors preoperatively if a biopsy can be safely performed.

Surgical management of PAIS includes endarterectomy, pulmonary artery resection with pneumonectomy or lobectomy, and pulmonary artery reconstruction. Blackmon et al. reported that the median survival time was 36.5 ± 20.2 months for patients who underwent curative resection compared with 11 ± 3 months for those who underwent incomplete tumor resection.^10)^ We considered performing the radical surgery and scheduled left pneumonectomy, accompanied by the resection of the main and right pulmonary arteries and reconstruction of the right pulmonary artery. We held a conference to establish the surgical strategy, such as performing either “one-stage or two-stage surgery” or maintaining “the same operative field or changing the operative field for pneumonectomy.” In general, resection of the main and right pulmonary arteries and reconstruction of the right pulmonary artery are performed via median sternotomy, whereas left pneumonectomy is performed via a posterolateral incision. Ichinokawa et al. reported that cardiovascular and lung surgeries could be performed on different days even in cases requiring pulmonary artery reconstruction.^11)^ The advantage of this approach is that a two-stage surgical approach helps ensure a clear surgical field of view, as CPB tends to cause bleeding in one-stage surgery.^11)^ However, in this case, we decided to perform the surgery on the same day via median sternotomy without changing the position. The 1st reason was to avoid delaying postoperative adjuvant therapy by performing surgery in two parts. Secondino et al. reported a trend toward better survival in patients with PAIS who received postoperative chemotherapy and/or radiation therapy than in those who underwent surgery alone.^12)^ The patient received chemoradiotherapy in the early postoperative period because of tumor cell positivity in the surgical margins and the possibility of microscopic metastasis preoperatively. The other reason was that we thought that we could safely perform pneumonectomy via median sternotomy by collapsing the heart under CPB. Generally, during pneumonectomy via median sternotomy, the inferior pulmonary vein is difficult to identify because the heart blocks its view.^13)^ Additional incisions and thoracoscopic assistance are occasionally required to manipulate the inferior pulmonary vein.^13)^ In this case, the left inferior pulmonary vein was safely manipulated by collapsing the heart under CPB, without the need for additional incisions. In situations where an adequate operative field cannot be achieved because of intrathoracic adhesions, we plan to utilize thoracoscopic assistance or make an additional incision. When no significant adhesions are anticipated, this surgical approach via median sternotomy is considered both appropriate and safe.

## CONCLUSIONS

We report the case of a patient who was diagnosed with PAIS by endovascular catheter biopsy and underwent left pneumonectomy accompanied by resection of the main and right pulmonary arteries and reconstruction of the right pulmonary artery via median sternotomy, performed by both a cardiovascular surgeon and a thoracic surgeon. Endovascular catheter biopsies are useful for differentiating thrombi from tumors. Furthermore, an early diagnosis of PAIS is important to ensure adequate time for discussing the surgical strategy with thoracic surgeons.

## DECLARATIONS

### Availability of data and materials

Data sharing does not apply to this article, as no datasets were generated or analyzed during the current study.
